# Marginal structural models for the estimation of the risk of Diabetes Mellitus in the presence of elevated depressive symptoms and antidepressant medication use in the Women’s Health Initiative observational and clinical trial cohorts

**DOI:** 10.1186/s12902-015-0049-7

**Published:** 2015-10-12

**Authors:** Christine Frisard, Xiangdong Gu, Brian Whitcomb, Yunsheng Ma, Penelope Pekow, Martha Zorn, Deidre Sepavich, Raji Balasubramanian

**Affiliations:** Division of Preventive and Behavioral Medicine, Department of Medicine, University of Massachusetts Medical School, 55 Lake Avenue North, Worcester, MA 01655 USA; Division of Biostatistics and Epidemiology, School of Public Health and Health Sciences, University of Massachusetts, 715 North Pleasant Street, Amherst, MA 01003 USA

**Keywords:** Antidepressant medication, Depression, Type 2 diabetes, Marginal structural models, Propensity score

## Abstract

**Background:**

We evaluate the combined effect of the presence of elevated depressive symptoms and antidepressant medication use with respect to risk of type 2 diabetes among approximately 120,000 women enrolled in the Women’s Health Initiative (WHI), and compare several different statistical models appropriate for causal inference in non-randomized settings.

**Methods:**

Data were analyzed for 52,326 women in the Women’s Health Initiative Clinical Trials (CT) Cohort and 68,169 women in the Observational Study (OS) Cohort after exclusions. We included follow-up to 2005, resulting in a median duration of 7.6 years of follow up after enrollment. Results from three multivariable Cox models were compared to those from marginal structural models that included time varying measures of antidepressant medication use, presence of elevated depressive symptoms and BMI, while adjusting for potential confounders including age, ethnicity, education, minutes of recreational physical activity per week, total energy intake, hormone therapy use, family history of diabetes and smoking status.

**Results:**

Our results are consistent with previous studies examining the relationship of antidepressant medication use and risk of type 2 diabetes. All models showed a significant increase in diabetes risk for those taking antidepressants. The Cox Proportional Hazards models using baseline covariates showed the lowest increase in risk , with hazard ratios of 1.19 (95 % CI 1.06 – 1.35) and 1.14 (95 % CI 1.01 – 1.30) in the OS and CT, respectively. Hazard ratios from marginal structural models comparing antidepressant users to non-users were 1.35 (95 % CI 1.21 – 1.51) and 1.27 (95 % CI 1.13 – 1.43) in the WHI OS and CT, respectively – however, differences among estimates from traditional Cox models and marginal structural models were not statistically significant in both cohorts. One explanation suggests that time-dependent confounding was not a substantial factor in these data, however other explanations exist. Unadjusted Cox Proportional Hazards models showed that women with elevated depressive symptoms had a significant increase in diabetes risk that remained after adjustment for confounders. However, this association missed the threshold for statistical significance in propensity score adjusted and marginal structural models.

**Conclusions:**

Results from the multiple approaches provide further evidence of an increase in risk of type 2 diabetes for those on antidepressants.

## Background

Diabetes is a chronic illness with serious health consequences, such as adult blindness, non-traumatic limb amputation, renal failure and neuropathy. Previous literature has noted considerable diabetes and depression among postmenopausal women, with a prevalence rate that is approximately 12 % for each [1, 2]. Recent literature also suggests an increased risk of diabetes among those who are depressed and on antidepressant medications [3 –6 ]. It is increasingly important to further investigate whether depression or antidepressant medication is influencing this association given that approximately 11 % of American women take antidepressant medication, and use is rising [7]. While the rates of use for depression treatment has remained the same, off-label use of antidepressants has increased significantly [8]. Examples of off-label use include treatment for certain types of pain, fibromyalgia, insomnia, and general unhappiness. In this analysis, we compare four statistical approaches to evaluate the combined effect of the presence of elevated depressive symptoms and antidepressant medication use on incident type 2 diabetes using data on approximately 120,000 women in the Women’s Health Initiative (WHI).

The WHI is a longitudinal study including repeated measurements for presence of elevated depressive symptoms, antidepressant use and self-reported diagnosis of type 2 diabetes. A previously reported analysis in the WHI [9] found that elevated depressive symptoms and antidepressant medication use resulted in increased type 2 diabetes risk. This analysis was based on Cox models that are subject to bias in the presence of time-dependent confounding [10–12]. A probable mechanism that gives rise to time-dependent confounding in this context is shown in Fig. 2 (Appendix) - participants with increased BMI could be more likely to be depressed and/or on antidepressants in the future [13]. Moreover, BMI and depression/antidepressant medication use can also significantly influence future diabetes risk. Thus, an analysis based on Cox models as in the previous literature [9] to evaluate the causal relationship between presence of elevated depressive symptoms/antidepressant use (exposure) and diabetes risk (outcome) could result in biased estimates by misattributing the effects of time-dependent confounders to the exposures of interest.

In the presence of time-dependent confounding, all Cox models including those that incorporate time-varying covariates are subject to bias [10–12]. Marginal structural models (MSMs) overcome limitations of Cox models - MSMs use inverse probability of treatment/exposure weighting and, given assumptions, can yield consistent and unbiased causal estimates of the effect of exposure [10–12]. Other methods in this setting include propensity score adjusted models in which the probability of treatment assignment conditional on baseline covariates (or propensity score) is adjusted for as an additional covariate.

The objective of this work was to estimate the combined effect of antidepressant medication use/presence of elevated depressive symptoms on type 2 diabetes in the WHI, and to compare results obtained from MSMs and propensity score adjusted Cox models to more traditional approaches such Cox models [9, 14, 15]. The results presented in this paper go beyond a previously reported analysis of this hypothesis in the WHI [9] by comparing results from four different statistical approaches including MSMs that are recommended for causal inference in observational studies.

## Methods

### Women’s health initiative (WHI)

The WHI enrolled 68,132 participants into clinical trials (WHI-CT) and 93,676 participants into an observational study (WHI-OS) between 1993 and 1998 [16–19]. Eligibility criteria included: postmenopausal women aged 50 to 79 years, reliable/mentally competent, and expected survival and local residency for at least three years. Medication use, presence of elevated depressive symptoms, and diabetes status were collected from participants over an average of 7.6 years of follow-up. We analyzed data with follow-up to 2005.

### Study variables

#### Incident diabetes

Diabetes status was assessed by self-report at baseline and at each annual follow-up visit, which has been found to be a reliable indicator of diagnosed diabetes [20]. Time to diabetes was calculated as the interval between study enrollment and development of diabetes as evidenced by an annual medical history update, or censorship (death or end of study participation).

#### Antidepressant medication use

WHI-CT participants were instructed to bring all current prescription and nonprescription medications in original containers to clinic visits at baseline and years 1, 3, 6, and 9. WHI-OS medication data were collected at baseline and year 3. The Master Drug Data Base (MDDB: Medi-Span, Indianapolis, IN) was used to categorize the medications. Based on the MDDB classification, a binary indicator for antidepressant medication use was created. Antidepressants include the following major groups: 1) Selective serotonin reuptake inhibitors; 2) Monoamine oxidase inhibitors; 3) Tricyclic antidepressants; 4) Tetracyclics; 5) Serotonin/norepinephrine reuptake inhibitors (SNRIs); 6) Aminoketones; 7) Triazolopyridines; and 8) Dibenzoxazepine. A dichotomous indicator of antidepressant medication use was then created for each measurement period [9]. Due to sample size limitations, we did not perform analyses by class of medication.

#### Elevated depressive symptoms

Elevated depressive symptoms were measured using the 6-item Center for Epidemiological Studies Depression Scale (CES-D) [21]. A participant was determined to have elevated depressive symptoms if their score was 5 or higher on the CES-D. Presence of elevated depressive symptoms was available at baseline and year 3 in the WHI-OS and at baseline, year 1, and close out in the WHI-CT. Only 6 % of participants were assessed at years 3, 6, and 9. Due to high levels of missing values at year 1 and later, analysis in the CT cohort adjusted only for baseline presence of elevated depressive symptoms.

#### BMI

BMI was available at baseline and year 3 in the WHI-OS and at baseline, years 1, 3, 6 and 9 in the WHI-CT.

#### Other covariates

Other variables available at baseline for inclusion in multivariable models include: age, race/ethnicity (American Indian/Alaskan Native; Asian or Pacific Islander; Black/African American; Hispanic/Latino; White; Other), education (<=high school; high school or GED; > = high school, but less than 4 years of college; 4 or more years of college), minutes of recreational physical activity per week, total energy intake, hormone therapy use (never, former, current), family history of diabetes (no, yes, don’t know) and smoking status (never smoked, past smoker, current smoker).

### Statistical analysis

#### Analysis datasets

**Fig. 1:**
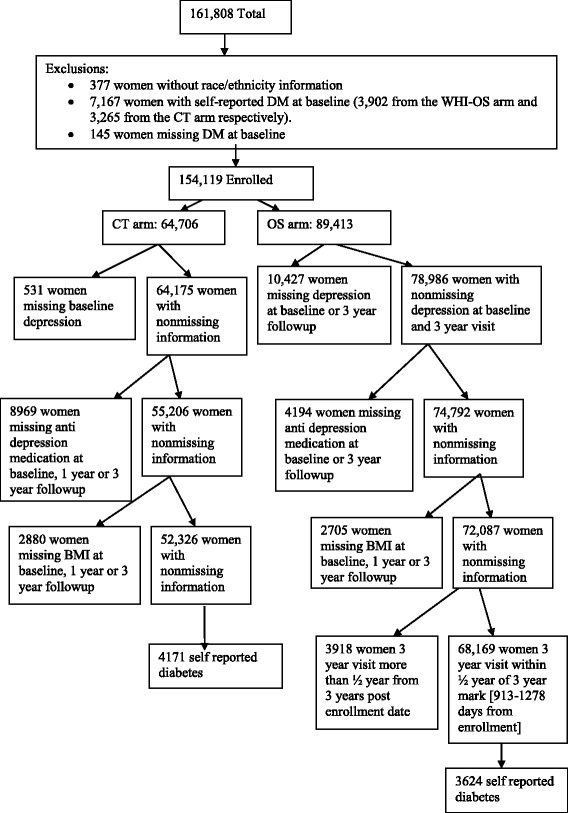
Flow chart describing analytic cohort included for the investigation (N = 120,495)

**WHI OS:** included 68,169 women after exclusions for self-reported diagnosis of diabetes at baseline or missing data on one or more of the following: baseline diabetes status, race/ethnicity, presence of elevated depressive symptoms, antidepressant medication use, BMI or women for whom the Year 3 visit occurred more than 3.5 years post enrollment (Fig. 1). 3624 (5.3 %) women reported diagnosis of type 2 diabetes during follow-up. Antidepressant use, BMI, presence of elevated depressive symptoms was available at baseline and year 3 and included in analyses.

**WHI CT:** included 52,326 women after the following exclusions - women with a self-reported diagnosis of diabetes at baseline or missing data on one or more of the following: diabetes status at baseline, race/ethnicity, baseline presence of elevated depressive symptoms, antidepressant medication use, BMI (Fig. 1). 4171 (8.0 %) women reported diagnosis of type 2 diabetes during follow-up. Antidepressant use and BMI were recorded at baseline, year 1 and year 3. Models adjusted for presence of elevated depressive symptoms at baseline because presence of elevated depressive symptoms was only measured on a small percentage (6.26 %) of women after year 1.

### Statistical models

We compared results from four approaches for estimating the association between the presence of elevated depressive symptoms/antidepressant medication use with incident type 2 diabetes, in the WHI-OS and WHI-CT datasets separately. Results reported by Ma and colleagues [9] were based on multivariable Cox models as in Approach 1 and Approach 2 described below. The results from these models were compared to propensity score adjusted models (Approach 3) and MSMs (Approach 4) [22–25]. The interaction of elevated depressive symptoms and antidepressant medication use was investigated in all models, but all interactions were insignificant (p > 0.26 in WHI-OS, p > 0.06 in WHI-CT).

**Approach 1** is a Cox proportional hazards (PH) model including the following covariates at baseline: elevated depressive symptoms, antidepressant use, BMI, age, ethnicity, education, minutes of recreational physical activity per week, total energy intake, hormone therapy use, family history of diabetes and smoking status.

**Approach 2** is a Cox model with time-varying values of elevated depressive symptoms (WHI-OS only), antidepressant medication use and BMI, and baseline values of age, ethnicity, education, minutes of recreational physical activity per week, total energy intake, hormone therapy use, family history of diabetes and smoking status.

**Approach 3** is similar to Approach 2, with additional adjustment for propensity for taking antidepressants at baseline. The propensity score was calculated to predict antidepressant use at baseline (outcome) from a logistic model. Predictors included in the model were baseline measures of BMI, age, ethnicity, education, minutes of recreational physical activity per week, total energy intake, hormone therapy use, family history of diabetes and smoking status.

**Approach 4** is a MSM for a time to event outcome [10]. Two separate logistic regression models were fit to calculate the probability of being on antidepressants and the probability of censoring (not observing the outcome by that point in time). The models incorporated time-varying data on the presence of elevated depressive symptoms and BMI while adjusting for baseline values of age, ethnicity, education, minutes of recreational physical activity per week, total energy intake, hormone therapy use, family history of diabetes and smoking status. These models were used to determine the stabilized Inverse Probability of Treatment (IPTW) weights. In the WHI-CT analysis, models were also adjusted for clinical site. To relax the linearity assumption, we added a quadratic function of time to the model. Model details and associated SAS code are included in the Appendix.

## Results

Table 1 presents baseline characteristics of our study population, by antidepressant medication use. Baseline characteristics by presence of elevated depressive symptoms are shown in Table 4 (Appendix). In both cohorts, the mean age was approximately 63 years old (63.6 (OS); 62.8 (CT)). Women were primarily White (86.5 % (OS); 84.4 % (CT)) and most had greater than high school education (80 % (OS); 76.4 % (CT)). Approximately 30 % of women on antidepressants had elevated depressive symptoms (28.2 % (OS); 29.8 % (CT)). A greater percentage of antidepressant medication users than non-users reported current use of hormone replacement therapy (64.9 % vs. 49.7 % (OS); 53.6 % vs. 36.3 % (CT)). Antidepressant users and non-users had approximately equal proportions that reported a family history of diabetes (30 % vs. 30.6 % (OS); 31.4 % vs. 31.6 % (CT)) or current smoking (7.2 % vs. 5.4 % (OS); 9.0 % vs. 7.2 % (CT)). Mean BMI was similar for those on antidepressants vs. not (27.91 vs. 26.70 (OS); 29.41 vs. 28.48 (CT)).

**Table 1:** Baseline characteristics of study participants in the Women’s Health Initiative (N = 120,495)

	Antidepressant medication use					
	No	Yes	Total	No	Yes	Total
	*N* (%)	*N* (%)	*N* (%)	N (%)	*N* (%)	*N* (%)
	WHI-OS N = 68,169			WHI-CT N = 52,326		
Baseline presence of elevated depressive symptoms^a^						
No	54923 (86.7 %)	3440 (71.8 %)	58363 (85.6 %)	42582 (86.7 %)	2238 (70.2 %)	44820 (85.7 %)
Yes	8457 (13.3 %)	1349 (28.2 %)	9806 (14.4 %)	6557 (13.3 %)	949 (29.8 %)	7506 (14.3 %)
Age^a^						
Mean (SD)	63.7 (7.3)	62.1 (7.3)	63.6 (7.3)	62.8 (6.9)	61.77 (6.9)	62.8 (6.9)
Median (IQR)	64.0 (11.0)	62.0 (12.0)	64.0 (11.0)	63.0 (11.0)	61.0 (11.0)	63.0 (11.0)
Ethnicity^a^						
American Indian/Alaskan Native	208 (0.3 %)	19 (0.4 %)	227 (0.3 %)	181 (0.4 %)	8 (0.3 %)	189 (0.4 %)
Asian or Pacific Islander	2009 (3.2 %)	44 (0.9 %)	2053 (3.0 %)	1207 (2.5 %)	22 (0.7 %)	1229 (2.4 %)
Black/African American	4037 (6.4 %)	158 (3.3 %)	4195 (6.2 %)	4266 (8.7 %)	119 (3.7 %)	4385 (8.4 %)
Hispanic/Latino	1845 (2.9 %)	146 (3.1 %)	1991 (2.9 %)	1711 (3.5 %)	85 (2.7 %)	1796 (3.4 %)
White	54614 (86.2 %)	4380 (91.5 %)	58994 (86.5 %)	41243 (83.9 %)	2924 (91.8 %)	44167 (84.4 %)
Other	667 (1.1 %)	42 (0.9 %)	709 (1.0 %)	531 (1.1 %)	29 (0.9 %)	560 (1.1 %)
Education						
<= High school	2409 (3.8 %)	189 (4 %)	2598 (3.8 %)	2302 (4.7 %)	144 (4.5 %)	2446 (4.7 %)
High school or GED	9765 (15.5 %)	752 (15.9 %)	10517 (15.5 %)	8991 (18.4 %)	587 (18.5 %)	9578 (18.4 %)
> = High school, but less than 4 years of college	22587 (35.9 %)	1765 (37.2 %)	24352 (36.0 %)	19110 (39.1 %)	1236 (39.0 %)	20346 (39.1 %)
4 or more years of college	28152 (44.7 %)	2036 (42.9 %)	30188 (44.6 %)	18426 (37.7 %)	1199 (37.9 %)	19625 (37.7 %)
BMI^a^						
Mean (SD)	26.7 (5.5)	27.9 (6.0)	26.8 (5.5)	28.5 (5.6)	29.4 (6.0)	28.5 (5.7)
Median (IQR)	25.6 (6.3)	26.7 (7.2)	25.7 (6.4)	27.6 (7.2)	28.4 (8.0)	27.6 (7.2)
Minutes of recreational physical activity per week^a^						
Mean (SD)	206.9 (186.2)	172.1 (174.8)	204.5 (185.6)	163.2 (169.0)	138.9 (159.2)	161.7 (168.5)
Median (IQR)	165.0 (225.0)	135.0 (212.5)	165.0 (225.0)	125.0 (215.0)	85.0 (195.0)	120.0 (215.0)
Total energy intake (kcal/day)^a^						
Mean (SD)	1533.1 (599.9)	1615.1 (640.4)	1538.8 (603.2)	1716.7 (679.5)	1819.7 (711.9)	1723.0 (682.0)
Median (IQR)	1461.3 (730.1)	1533.2 (765.8)	1465.4 (733.4)	1618.3 (832.5)	1716.3 (870.8)	1623.7 (833.7)
Hormone replacement therapy use^a^						
Never	18423 (29.6 %)	738 (15.7 %)	19161 (28.6 %)	17793 (37.9 %)	662 (21.6 %)	18455 (36.9 %)
Former	12885 (20.7 %)	914 (19.4 %)	13799 (20.6 %)	12122 (25.8 %)	760 (24.8 %)	12882 (25.7 %)
Current	30931 (49.7 %)	3055 (64.9 %)	33986 (50.8 %)	17057 (36.3 %)	1644 (53.6 %)	18701 (37.4 %)
Family history of diabetes						
No	41656 (66.0 %)	3114 (65.2 %)	44770 (65.9 %)	31282 (63.9 %)	2022 (63.7 %)	33304 (63.9 %)
Yes	18923 (30.0 %)	1463 (30.6 %)	20386 (30.0 %)	15378 (31.4 %)	1004 (31.6 %)	16382 (31.4 %)
Don’t know	2569 (4.1 %)	198 (4.1 %)	2767 (4.1 %)	2280 (4.7 %)	147 (4.6 %)	2427 (4.7 %)
Smoking status^a^						
Never smoked	32614 (52.0 %)	2188 (46.2 %)	34802 (51.6 %)	25547 (52.5 %)	1429 (45.2 %)	26976 (52.1 %)
Past smoker	26685 (42.6 %)	2207 (46.6 %)	28892 (42.9 %)	19615 (40.3 %)	1445 (45.7 %)	21060 (40.6 %)
Current smoker	3370 (5.4 %)	339 (7.2 %)	3709 (5.5 %)	3495 (7.2 %)	285 (9.0 %)	3780 (7.3 %)
Reported diagnosis of diabetes by end of follow-up^a^						
No	60077 (94.8 %)	4468 (93.3 %)	64545 (94.7 %)	45269 (92.1 %)	2886 (90.6 %)	48155 (92.0 %)
Yes	3303 (5.2 %)	321 (6.7 %)	3624 (5.3 %)	3870 (7.9 %)	301 (9.4 %)	4171 (8.0 %)
Years from enrollment to developing diabetes^a^						
Mean (SD)	7.7 (1.5)	7.6 (1.5)	7.7 (1.5)	8.0 (1.7)	7.9 (1.7)	8.0 (1.7)
Median (IQR)	7.9 (2.0)	7.9 (2.0)	7.9 (2.0)	8.0 (1.7)	8.00 (1.9)	8.0 (1.8)

^a^ *P* – value of <0.002 or lower

### OS cohort

#### Elevated depressive symptoms

An unadjusted model including only baseline values of antidepressant medication use and presence of elevated depressive symptoms showed a significant increase in diabetes risk for those with elevated depressive symptoms (HR 1.34; 95 % CI: 1.23-1.45). Approaches 1 and 2 that adjust for other confounders also resulted in a statistically significant association between the presence of elevated depressive symptoms and increased risk of diabetes – however, this association did not remain statistically significant in propensity score adjusted models and MSMs.

#### Antidepressant medication use

All models showed a consistent, statistically significant increase in diabetes risk for those exposed to antidepressant medications vs. those who were not (Table 2). Approach 1 using baseline measures only yielded a HR of 1.19 (95 % CI 1.06 – 1.35). Approach 2 and Approach 3 using time-varying antidepressant medication use, presence of elevated depressive symptoms and BMI, yielded almost identical results for antidepressant medication use [Approach 2: 1.31 (95 % CI 1.18 – 1.46); Approach 3: 1.32 (95 % CI 1.19 – 1.47)]. There were no significant differences in the HRs estimated for the presence of elevated depressive symptoms between Approaches 2 and 3. The results from MSMs (Approach 4) were almost identical to Approaches 2 and 3 –the HR (CI) for antidepressant medication use was 1.35 (95 % CI 1.21 – 1.51). In this application, the MSM approach yielded similar results to the traditional extended Cox model.

**Table 2:** Hazard ratios for presence of elevated depressive symptoms and antidepressant use with respect to incident diabetes risk

	Unadjusted Cox PH model	Approach 1^a^	Approach 2^a^	Approach 3^a^	Approach 4^a^
	Cox PH Model (baseline antidepressant medication use and baseline presence of elevated depressive symptoms)	Cox PH Models (baseline values of all predictors)	Cox Models (time varying antidepressant medication use, presence of elevated depressive symptoms, and BMI; baseline values for other covariates)	Cox Models (time varying antidepressant medication use, presence of elevated depressive symptoms, and BMI; adjusted for propensity score and baseline values for other covariates)	Marginal Structural Models (time varying antidepressant medication use, presence of elevated depressive symptoms, and BMI; baseline values for other covariates)
Predictor	HR (95 % CI)	HR (95 % CI)	HR (95 % CI)	HR (95 % CI)	HR (95 % CI)
	Observational Cohort (OS) (*N* = 68,169)				
Antidepressant medication use up to year 3	1.26	1.19	1.31	1.32	1.35
	(1.12–1.41)	(1.06–1.35)	(1.18–1.46)	(1.19–1.47)	(1.21–1.51)
Presence of elevated depressive symptoms	1.34	1.11	1.12	1.09	1.10
	(1.23–1.45)	(1.02–1.21)	(1.03–1.23)	(1.00–1.19)	(1.00–1.20)
	Clinical Trial (CT) (*N* = 52,326)				
Antidepressant medication use up to year 3	1.17	1.14	1.26	1.25	1.27
	(1.04–1.31)	(1.01–1.30)	(1.12–1.41)	(1.12–1.40)	(1.13–1.43)
Presence of elevated depressive symptoms	1.31	1.13	1.12	1.09	1.10
	(1.21–1.42)	(1.04–1.23)	(1.03–1.22)	(0.996–1.18)	(1.00–1.20)

^a^Models adjusted for baseline values of age, ethnicity, education, minutes of recreational physical activity per week, total energy intake, hormone therapy use, family history of diabetes and smoking status

### CT cohort

#### Elevated depressive symptoms

As in the WHI-OS, the unadjusted model showed a significant increase in diabetes risk for those with elevated depressive symptoms. While this association was statistically significant in Approaches 1 and 2, it did not remain so in the propensity score adjusted model (Approach 3) and the MSM (Approach 4).

#### Antidepressant medication use

As in the WHI OS, all models in the WHI-CT showed a consistent, statistically significant increase in diabetes risk for those exposed to antidepressant medications vs. those who were not.

Table 3 presents an estimate of variation in antidepressant medication use and presence of elevated depressive symptoms at baseline and year 3, by cohort. In the WHI-OS, 4.9 % of women were using antidepressant medication at both time-points, whereas 88.5 % never used them. 2.2 % of women who were using antidepressant medication at baseline had stopped by year 3, and 4.5 % who were not using antidepressant medication at baseline had started by year 3. 6.4 % experienced elevated depressive symptoms at both baseline and year 3, while 76.3 % never did. 8 % of women who experienced elevated depressive symptoms at baseline did not report experiencing those symptoms at year 3, and 9.3 % without elevated depressive symptoms at baseline did experience them by year 3. Similar patterns were observed in the WHI-CT.

**Table 3:** Estimate of variation in presence of depressive symptoms and antidepressant medication use over time

	WHI-OS *N* = 68,169	
	Antidepressant use at baseline	No antidepressant use at baseline
	*N* (%)	*N* (%)
Antidepressant use at year 3	3321 (4.9 %)	3083 (4.5 %)
No antidepressant use at year 3	1468 (2.2 %)	60297 (88.5 %)
	Presence of elevated symptoms at baseline	No presence of elevated symptoms at baseline
	*N* (%)	*N* (%)
Presence of elevated symptoms at year 3	4348 (6.4 %)	6333 (9.3 %)
No presence of elevated symptoms at year 3	5458 (8.0 %)	52030 (76.3 %)
	Baseline	Year 3
	Mean (SD)	Mean (SD)
BMI	26.80 (5.5)	27.09 (5.6)
	WHI-CT *N* = 52,326	
	Antidepressant use at baseline	No antidepressant use at baseline
	*N* (%)	*N* (%)
Antidepressant use at year 3	2031 (3.9 %)	1979 (3.8 %)
No antidepressant use at year 3	1156 (2.2 %)	47160 (90.1 %)
	Baseline	Year 3
	Mean (SD)	Mean (SD)
BMI	28.54 (5.7)	28.78 (5.9)

Table 5 (Appendix) presents the HRs and 95 % CIs for all covariates in the MSMs, in the WHI-OS and CT cohorts. Table 6 (Appendix) presents the distributions of the IPTW weights, including the estimated probability of having one’s own observed treatment history and censoring history at follow-up time points. The probability of remaining uncensored was close to 1 for both cohorts at each follow-up time point given both the baseline and time-varying covariates. There was variation in the probability of having one’s own observed treatment history, but the mean and median were close to 1 at 36 month follow-up in the WHI-OS and 12 and 36 month follow-up in the WHI-CT.

## Discussion

Previous research has shown that the prevalence of elevated depressive symptoms and diabetes is high in postmenopausal women [1, 26]. Ma and colleagues [9] found an increased risk of type 2 diabetes among women in the WHI cohorts who reported elevated depressive symptoms (HR 1.13 [95 % CI 1.07–1.20]) and antidepressant use at baseline (1.18 [95 % CI 1.10–1.28]), based on Cox models. Multivariable longitudinal analyses confirmed this relationship with recent antidepressant medication use, but found only prolonged elevated depressive symptoms to be significantly associated with increased risk. Our analyses were performed on 120,495 women in the WHI and adjusted for the same confounders, but utilized Cox models as well as propensity score adjusted models and MSMs. Our analyses found a consistent, significant increase in diabetes risk among those reporting antidepressant medication use by four different statistical approaches. In all approaches considered, presence of elevated depressive symptoms was rendered marginally or non-significant after adjusting for confounders. Our results are consistent with previous studies examining the relationship of antidepressant medication use and risk of type 2 diabetes [3–6, 27, 28].

**Fig. 2:**
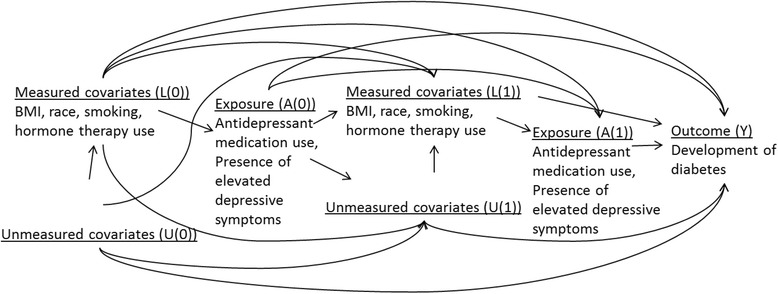
Illustration of time-dependent confounding by BMI in the association of antidepressant medication use and time to development of diabetes. Illustrates the hypothesis that there is time-varying confounding by BMI with regard to the association between diabetes risk and the presence of elevated depressive symptoms/antidepressant use. Let A denote the exposure or presence of elevated depressive symptoms/antidepressant use, L denotes measured covariates such as BMI or race, U denotes unmeasured covariates and Y denotes the outcome (diabetes). The causal graph illustrates that the probabilities of elevated depressive symptoms/antidepressant medication use (A) depends on BMI (L), but not U. There is confounding by measured covariates, but no confounding by unmeasured covariates. The probabilities of elevated depressive symptoms/antidepressant medication use at baseline (A(0)) is influenced by baseline BMI (L(0)). In our example, confounding is time dependent because exposure at time 1 (A(1)) is affected by previous exposure (A(0)) and BMI at time 1 (L(1))

Of the four different modeling approaches considered in this paper, MSMs are the gold standard for use in observational studies in which the presence of time-dependent confounding (Fig. 2, Appendix) is a possibility. For observational study settings in the absence of time-dependent confounding, propensity score adjusted models can be used to adjust for bias in exposure assignment. Propensity score adjusted models correct for confounding by indication – this bias is present in studies in which individuals who are prescribed or take a medication are inherently different in their risk profile with respect to outcome when compared to those who do not take the drug. In the absence of confounding by indication and time-dependent confounding, simpler Cox models with or without time-varying covariates are appropriate.

We did not observe differences in hazard ratio estimates between the four modeling approaches that were considered. One explanation for the concordance of estimates from these different approaches suggests that time-dependent confounding by BMI was not a substantial factor in these data – thus, BMI measured over the course of observation may not be strongly affected by exposure (i.e., as an intermediate) and/or did not exert a strong influence on our exposure and outcome of interest (i.e., as a confounder). However, other potential explanations for this observed concordance of results include limited longitudinal measurements of the key exposure variables, measurement error of the confounder and/or incorrect model specification of the dose response relationship with respect to the effects of the confounder on both exposure and outcome. However, this study was not designed to pinpoint the specific factor that caused the concordance of estimates from the various models.

A limitation of this work is that we had limited longitudinal follow up. The WHI-OS had two repeated measures, at baseline and at year 3. The WHI-CT had more repeated measures available, but we were only able to utilize three time points (baseline, year 1, year 3) due to high levels of missing data at later time points. In addition, in the WHI-CT, because presence of elevated depressive symptoms was measured on only a small percentage of participants after year 1, our models could not incorporate this factor as a time varying exposure. Due to limitations of the available data, the analysis did not account for antidepressant dose and adherence. While a bidirectional association between depression and diabetes risk is biologically plausible, our study was not designed to tease out the direction of association. Lastly, due to cost considerations, diabetes status was ascertained through self-reported questionnaires – this could result in modest levels of outcome misclassification. Statistical models that account for the error-prone self-reported outcomes would be useful in this context.

## Conclusions

Our analyses provide further evidence that a significant increase in diabetes risk is observed for those on antidepressant medications in the WHI. In addition, our results comparing modeling approaches demonstrates that in some settings, results from more complex methods such as MSMs may not differ substantially from traditional methods of analysis – however, we recommend that these methods be explored to establish the validity of initial findings.

## Appendix

SAS Code to Fit the Marginal Structural Cox Proportional Hazards Model

Here we provide details on how to set up the data, along with SAS code to perform the analyses in Approach 4 using Marginal Structural Models for a time to event outcome. The first part of the analysis uses the proc logistic procedure to calculate the probability of not being on antidepressants (Models 1 and 2). Data in the file ‘iptw’ contains 1 record per subject for each time point included in the analysis. For example, in the WHI-OS dataset, we have measurements at baseline and year 3. Each subject has two records, one for baseline and one for year 3. Next we use the proc logistic procedure to calculate the probability of not being censored (Models 3 and 4). In the data file ‘iptw2’ the data has been expanded so that a subject has 1 record per month until her time to diabetes or time to censoring. The dataset ‘main’ merges Models 1-4 together to calculate the IPTW weights. This again is an expanded dataset so a subject has 1 record per month until her time to diabetes or time to censoring. The dataset ‘main_w’ contains the inverse probability of treatment weights and is the dataset used to run the marginal structural model analysis. Last, we use the proc genmod procedure to fit the final pooled logistic regression model to obtain the estimates of our association of interest.

Antidep is a dichotomous 0/1 indicator of the participant being on or off antidepressants. Model 1 includes that dichotomous indicator as the outcome, a time-dependent intercept and baseline values of the following covariates as predictors: presence of elevated depressive symptoms (base_depression), BMI (base_bmi), Age (age), Ethnicity (ethnic), Education (educ4), Minutes of physical activity per week (tminwk), Total energy intake (base_energy_cat), Family history of diabetes (diabrel), Hormone therapy use (hormstat) and smoking status (smoking). Model 2 mirrors Model 1 with the addition of the most recent time-dependent values of the following covariates: presence of elevated depressive symptoms (depression), and BMI (bmi).

The outcome variable in Models 3 and 4 is a dichotomous indicator of whether or not the participant has been censored up to that time point. Model 3 includes as regressors the baseline values for the following covariates: the dichotomous indicator of antidepressant medication use (antidep), presence of elevated depressive symptoms (base_depression), BMI (base_bmi), Age (age), Ethnicity (ethnic), Education (educ4), Minutes of physical activity per week (tminwk), Total energy intake (base_energy_cat), Family history of diabetes (diabrel), Hormone therapy use (hormstat) and smoking status (smoking). All available person months are included. Model 4 mirrors Model 3 with the inclusion of the most recent time-dependent value of the following covariates: antidepressant medication use (antidepressant_tv), presence of elevated depressive symptoms (depression) and BMI (bmi). Models 3 and 4 also include variables for ‘month’ and ‘month^2^’ to relax the linearity assumption.

We merge Models 1-4 together and in the following data step use the predicted values from those models to compute our IPTW estimates, denoted by stabw and nstabw for the stabilized and non-stabilized weights, respectively.

In our last step, we use the proc genmod procedure to fit the weighted pooled logistic model to obtain estimates of our association of interest from our Marginal Structural Cox PH Model. The outcome here is diabetes, which is a dichotomous indicator of whether or not the participant developed diabetes during that month. The patient ID variable and the independent working correlation matrix (subject = id/type = ind) must be specified. We weighted the model used the stabilized weights by using the ‘scwgt stabw’ statement in the procedure. The ‘estimate’ statement asks the procedure to report the odds ratio for our main associations of interest, in addition to the coefficients in the model.

/*calculate probability of not being on antidepressants*/

/* Model 1*/

**proc sort**data=iptw;

by id month;

**run**;

**proc logistic**data=iptw;

class base_depression ethnic educ4 base_energy_cat hormstat smoking diabrel;

where month=**36**;

model antidep=base_depression base_bmi age ethnic educ4 tminwk base_energy_cat diabrel hormstat smoking month;

output out=model1 p=pandep_0_temp;

**run**;

/* Model 2*/

**proc logistic**data=iptw;

where month=**36**;

class base_depression depression ethnic educ4 base_energy_cat energy_cat hormstat smoking diabrel;

model antidep=base_depression depression base_bmi bmi age ethnic educ4 tminwk base_energy_cat diabrel hormstat smoking month;

output out=model2 p=pandep_w_temp;

**run**;

/*calculate probability of not being censored*/

/* Model 3*/

**proc logistic**data=iptw2;

class antidep depression ethnic educ4 base_energy_cat hormstat smoking diabrel;

model censor_tv=antidep depression bmi age ethnic educ4 tminwk base_energy_cat diabrel hormstat smoking month month_sq;

output out=model3 p=punc_0;

**run**;

/* Model 4*/

**proc logistic**data=iptw2;

class antidep antidepressant_tv depression depression_tv ethnic educ4 base_energy_cat hormstat smoking diabrel;

model censor_tv=antidep antidepressant_tv depression bmi age ethnic educ4 tminwk base_energy_cat diabrel hormstat smoking depression_tv bmi_tv month month_sq;

output out=model4 p=punc_w;

**run**;

/*merge data*/

**data**main;

merge temp1 temp2 model3(in=a) model4(in=b);

by id month; if a=**1**and b=**1**;

if first.id then do;

pandep_0 =**1**;

pandep_w =**1**;

end;

if month=**36**then do;

pandep_0=pandep_0_temp;

pandep_w=pandep_w_temp;

end;

retain pandep_0 pandep_w;

**run**;

/* Calculate the weights*/

**data**main_w;

set main;

by id month;

/*reset variables for a new patient*/

if first.id then do;

k2_0=**1**; k2_w=**1**;

end;

retain k2_0 k2_w;

/*inverse probability of censoring weights*/

else do;

k2_0=k2_0*punc_0;

k2_w=k2_w*punc_w;

end;

/* Inverse probability of treatment weights */

if antidepressant_tv=**0**and month>=**36**then k1_0=pandep_0;

if antidepressant_tv=**0**and month>=**36**then k1_w=pandep_w;

if antidepressant_tv=**1**and month>=**36**then k1_0=(**1**-pandep_0);

if antidepressant_tv=**1**and month>=**36**then k1_w=(**1**-pandep_w);

if month<**36**then k1_0=**1**;

if month<**36**then k1_w=**1**;

/* Stabilized and non stabilized weights */

stabw=(k1_0*k2_0)/(k1_w*k2_w);

nstabw=**1**/(k1_w*k2_w);

**run**;

/* Pooled logistic regression model to run the MSM analysis */

**proc genmod**data=main_w descending;

class id ethnic educ4 hormstat smoking base_energy_cat diabrel;

model diabetes_tv=antidepressant_tv ethnic educ4 tminwk diabrel hormstat smoking depression_tv bmi_tv age base_energy_cat month month_sq/ link=logit dist=bin;

scwgt stabw;

repeated subject=id/ type=ind;

estimate "log O.R. antidepressant" antidepressant_tv**1**/ exp;

estimate "log O.R. depressive symptoms" depression_tv**1**/ exp;

**run**;

**Table 4:** Baseline characteristics of study participants, by presence of elevated depressive symptoms

	Presence of elevated depressive symptoms					
	No	Yes	Total	No	Yes	Total
	*N* (%)	*N* (%)	*N* (%)	*N* (%)	*N* (%)	*N* (%)
	WHI-OS *N* = 68,169			WHI-CT *N* = 52,326		
Antidepression medication use^a^						
No	54923 (94.1 %)	8457 (86.2 %)	63380 (93.0 %)	42582 (95.0 %)	6557 (87.4 %)	49139 (93.9 %)
Yes	3440 (5.9 %)	1349 (13.8 %)	4789 (7.0 %)	2238 (5.0 %)	949 (12.6 %)	3187 (6.1 %)
Age^a^						
Mean(SD)	63.7 (7.2)	62.9 (7.5)	63.6 (7.3)	62.9 (6.9)	61.8 (7.1)	62.8 (6.9)
Median(IQR)	64.0 (11.0)	63.0 (12.0)	64.0 (11.0)	63.0 (11.0)	61.0 (11.0)	63.0 (11.0)
Ethnicity^a^						
American Indian/Alaskan Native	172 (0.3 %)	55 (0.6 %)	227 (0.3 %)	151 (0.3 %)	38 (0.5 %)	189 (0.4 %)
Asian or Pacific Islander	1839 (3.2 %)	214 (2.2 %)	2053 (3.0 %)	1124 (2.5 %)	105 (1.4 %)	1229 (2.4 %)
Black/African American	3490 (6.0 %)	705 (7.2 %)	4195 (6.2 %)	3607 (8.1 %)	778 (10.4 %)	4385 (8.4 %)
Hispanic/Latino	1478 (2.5 %)	513 (5.2 %)	1991 (2.9 %)	1313 (2.9 %)	483 (6.4 %)	1796 (3.4 %)
White	50798 (87 %)	8196 (83.6 %)	58994 (86.5 %)	38158 (85.1 %)	6009 (80.1 %)	44167 (84.4 %)
Other	586 (1.0 %)	123 (1.3 %)	709 (1.0 %)	467 (1.0 %)	93 (1.2 %)	560 (1.1 %)
Education^a^						
<= High school	1957 (3.4 %)	641 (6.6 %)	2598 (3.8 %)	1837 (4.1 %)	609 (8.2 %)	2446 (4.7 %)
High school or GED	8784 (15.2 %)	1733 (17.8 %)	10517 (15.5 %)	8074 (18.1 %)	1504 (20.2 %)	9578 (18.4 %)
> = High school, but less than 4 years of college	20643 (35.6 %)	3709 (38.2 %)	24352 (36.0 %)	17315 (38.9 %)	3031 (40.7 %)	20346 (39.1 %)
4 or more years of college	26557 (45.8 %)	3631 (37.4 %)	30188 (44.6 %)	17330 (38.9 %)	2295 (30.9 %)	19625 (37.7 %)
BMI^a^						
Mean(SD)	26.7 (5.4)	27.6 (6.0)	26.8 (5.5)	28.4 (5.6)	29.3 (6.0)	28.5 (5.7)
Median(IQR)	25.6 (6.3)	26.5 (7.1)	25.7 (6.4)	27.5 (7.1)	28.4 (7.7)	27.6 (7.2)
Minutes of recreational physical activity per week^a^						
Mean(SD)	210.1 (186.6)	170.8 (175.7)	204.5 (185.6)	165.4 (168.7)	139.7 (165.5)	161.7 (168.5)
Median(IQR)	170.0 (225.0)	127.5 (220.0)	165.0 (225.0)	125.0 (220.0)	85.0 (200.0)	120.0 (215.0)
Total energy intake (kcal/day)^a^						
Mean(SD)	1529.7 (588.9)	1593.2 (679.5)	1538.8 (603.2)	1710.7 (666.7)	1796.8 (762.7)	1723.0 (682.0)
Median(IQR)	1461.9 (719.7)	1493.9 (817.1)	1465.4 (733.4)	1618.0 (815.0)	1671.6 (951.6)	1623.7 (833.7)
HRT use ever^a^						
Never	16498 (28.8 %)	2663 (27.7 %)	19161 (28.6 %)	15945 (37.2 %)	2510 (34.9 %)	18455 (36.9 %)
Former	11590 (20.2 %)	2209 (22.9 %)	13799 (20.6 %)	10805 (25.2 %)	2077 (28.9 %)	12882 (25.7 %)
Current	29228 (51.0 %)	4758 (49.4 %)	33986 (50.8 %)	16099 (37.6 %)	2602 (36.2 %)	18701 (37.4 %)
Relative had adult diabetes^a^						
No	38718 (66.6 %)	6052 (62.0 %)	44770 (65.9 %)	28802 (64.5 %)	4502 (60.3 %)	33304 (63.9 %)
Yes	17225 (29.6 %)	3161 (32.4 %)	20386 (30.0 %)	13867 (31.1 %)	2515 (33.7 %)	16382 (31.4 %)
Smoking status^a^						
Never Smoked	29984 (52.0 %)	4818 (49.7 %)	34802 (51.6 %)	23382 (52.7 %)	3594 (48.4 %)	26976 (52.1 %)
Past Smoker	24751 (42.9 %)	4141 (42.7 %)	28892 (42.9 %)	18012 (40.6 %)	3048 (41.1 %)	21060 (40.6 %)
Current Smoker	2971 (5.1 %)	738 (7.6 %)	3709 (5.5 %)	3004 (6.8 %)	776 (10.5 %)	3780 (7.3 %)
Reported diagnosis of diabetes by end of follow-up^a^						
No	55407 (94.9%)	9138 (93.2%)	64545 (94.7%)	41399 (92.4%)	6756 (90.0%)	48155 (92.0%)
Yes	2956 (5.1%)	668 (6.8%)	3624 (5.3%)	3421 (7.6%)	750 (10.0%)	4171 (8.0%)
Years from enrollment to development of diabetes^a^						
Mean(SD)	7.7 (1.5)	7.6 (1.6)	7.7 (1.5)	8.0 (1.7)	7.9 (1.8)	8.0 (1.7)
Median(IQR)	7.9 (2.0)	7.9 (2.0)	7.9 (2.0)	8.0 (1.7)	8.0 (1.9)	8.0 (1.8)
Don’t Know	2212 (3.8 %)	555 (5.7 %)	2767 (4.1 %)	1975 (4.4 %)	452 (6.1 %)	2427 (4.7 %)

^a^ *P*-value <0.007 or lower

**Table 5:** Inverse probability of treatment-weighted estimates of the marginal structural model

Parameter	Estimate	SE	95 % Confidence limits		HR (95 % CI)	*P*-Value
WHI-OS						
Antidepressant medication use						<.0001
Yes	0.30	0.06	0.19	0.41	1.35 (1.21-1.51)	
No	0.00	0.00	0.00	0.00	1	.
Presence of elevated depressive symptoms						0.04
Yes	0.09	0.05	0.00	0.18	1.09 (1.00-1.20)	
No	0.00	0.00	0.00	0.00	1	.
Ethnicity						
American Indian/Alaskan Native	0.72	0.20	0.32	1.12	2.05 (1.38-3.06)	0.0004
Asian or Pacific Islander	0.78	0.09	0.61	0.95	2.18 (1.84-2.59)	<.0001
Black/African American	0.56	0.06	0.46	0.67	1.75 (1.58-1.95)	<.0001
Hispanic/Latino	0.52	0.09	0.35	0.69	1.68 (1.42-1.99)	<.0001
White	0.00	0.00	0.00	0.00	1	.
Other	0.35	0.15	0.05	0.65	1.42 (1.05-1.92)	0.02
Education level						
<= high school	0.37	0.08	0.22	0.52	1.45 (1.25-1.68)	<.0001
High school or GED	0.29	0.05	0.19	0.39	1.34 (1.21-1.48)	<.0001
> = High school, but less than 4 years of college	0.22	0.04	0.14	0.30	1.25 (1.15-1.35)	<.0001
4 or more years of college	0.00	0.00	0.00	0.00	1	.
Minutes or recreational physical activity per week	-0.0006	0.0001	-0.0008	-0.0004	0.9994 (0.9992-0.9996)	<.0001
Family history of diabetes						
No	-0.78	0.04	-0.85	-0.71	0.46 (0.43-0.49)	<.0001
Yes	0.00	0.00	0.00	0.00	1	.
Don’t know	-0.41	0.08	-0.57	-0.24	0.66 (0.57-0.79)	<.0001
Hormone therapy use						
Never	0.00	0.00	0.00	0.00	1	.
Former	0.01	0.05	-0.09	0.10	1.01 (0.91-1.11)	0.89
Current	-0.12	0.04	-0.20	-0.04	0.89 (0.82-0.96)	0.004
Smoking status						
Never smoked	0.00	0.00	0.00	0.00	1	.
Past smoker	0.05	0.04	-0.03	0.12	1.05 (0.97-1.13)	0.21
Current smoker	0.17	0.07	0.03	0.31	1.19 (1.03-1.36)	0.02
BMI	0.08	0.00	0.08	0.08	1.08 (1.08-1.09)	<.0001
Age	0.02	0.00	0.01	0.02	1.02 (1.01-1.02)	<.0001
Baseline total energy intake						
4.87- 1100.54	-0.21	0.05	-0.30	-0.11	0.81 (0.74-0.90)	<.0001
1100.55- 1437.42	-0.24	0.05	-0.33	-0.14	0.79 (0.72-0.87)	<.0001
1437.44- 1828.01	-0.18	0.05	-0.27	-0.08	0.84 (0.76-0.92)	0.00
> = 1828.02	0.00	0.00	0.00	0.00	1	.
WHI-CT						
Antidepressant medication use						<.0001
Yes	0.24	0.06	0.13	0.36	1.27 (1.14-1.43)	
No	0.00	0.00	0.00	0.00	1	.
Presence of elevated depressive symptoms						0.04
Yes	0.09	0.05	0.00	0.18	1.09 (1.00-1.20)	
No	0.00	0.00	0.00	0.00	1	.
Ethnicity						
American Indian/Alaskan Native	0.09	0.26	-0.41	0.60	1.09 (0.66-1.82)	0.71
Asian or Pacific Islander	0.73	0.10	0.54	0.91	2.08 (1.72-2.48)	<.0001
Black/African American	0.41	0.05	0.32	0.51	1.51 (1.38-1.67)	<.0001
Hispanic/Latino	0.50	0.08	0.35	0.65	1.65 (1.42-1.92)	<.0001
White	0.00	0.00	0.00	0.00	1	.
Other	0.38	0.14	0.10	0.65	1.46 (1.11-1.92)	0.007
Education						
<= high school	0.32	0.07	0.18	0.46	1.38 (1.20-1.58)	<.0001
High school or GED	0.20	0.05	0.10	0.29	1.22 (1.11-1.34)	<.0001
> = High school, but less than 4 years of college	0.22	0.04	0.14	0.30	1.25 (1.15-1.35)	<.0001
4 or more years of college	0.00	0.00	0.00	0.00	1	.
Minutes or recreational physical activity per week	-0.001	0.000	-0.001	0.000	0.999 (0.999-1.000)	<.0001
Family history of diabetes						
Yes	0.00	0.00	0.00	0.00	1	.
No	-0.68	0.03	-0.75	-0.61	0.51 (0.47-0.54)	<.0001
Don’t know	-0.32	0.07	-0.47	-0.18	0.73 (0.63-0.84)	<.0001
Hormone therapy use						
Never	0.00	0.00	0.00	0.00	1	.
Former	-0.002	0.04	-0.08	0.08	0.998 (0.92-1.08)	0.97
Current	-0.17	0.05	-0.26	-0.09	0.84 (0.77-0.91)	0.0001
Smoking status						
Never smoked	0.00	0.00	0.00	0.00	1	.
Past smoker	-0.01	0.04	-0.08	0.06	0.99 (0.92-1.06)	0.78
Current smoker	0.19	0.06	0.07	0.32	1.21 (1.07-1.38)	0.002
BMI	0.07	0.00	0.07	0.08	1.07 (1.07-1.08)	<.0001
Age	0.02	0.00	0.01	0.02	1.02 (1.01-1.02)	<.0001
Baseline total energy intake						
4.87- 1100.54	-0.04	0.05	-0.13	0.06	0.96 (0.88-1.06)	0.46
1100.55- 1437.42	-0.04	0.05	-0.13	0.05	0.96 (0.88-1.05)	0.43
1437.44- 1828.01	-0.06	0.04	-0.15	0.03	0.94 (0.86-1.03)	0.17
1828.02- 23020.93	0	0	0	0	1	.
Participated in hormone therapy trial						0.07
Yes	0.09	0.05	-0.01	0.20	1.09 (0.99-1.22)	
No	0	0	0	0	1	.
Participated in dietary modification trial						0.07
Yes	0.10	0.05	-0.01	0.20	1.11 (0.99-1.22)	
No	0	0	0	0	1	.
Participated in calcium/ vitamin D supplementation						0.44
Yes	-0.03	0.03	-0.09	0.04	0.97 (0.91-1.04)	
No	0	0	0	0	1	.

**Table 6:** Estimated probability of having one’s own observed treatment history and censoring history at follow-up

	*N*	Mean	SD	Median	Quartile range	Minimum	Maximum
WHI-OS							
36 months							
Probability of having observed antidepressant medication history							
Given baseline covariates	64048	0.91	0.05	0.91	0.06	0.49	0.99
Given time-varying covariates	64048	0.91	0.06	0.92	0.06	0.36	0.99
Probability of being uncensored							
Given baseline covariates	64099	0.99983	0.0000271	0.9998275	0.0000380	0.99963	0.99988
Given time-varying covariates	64099	0.99983	0.0000273	0.9998275	0.0000381	0.99964	0.99988
WHI-CT							
12 months							
Probability of having observed antidepressant medication history							
Given baseline covariates	46084	0.93	0.05	0.94	0.05	0.52	0.995
Given time-varying covariates	46084	0.93	0.05	0.94	0.05	0.30	0.996
Probability of being uncensored							
Given baseline covariates	46084	0.9999997	6.7564221E-8	0.9999997	9.1169629E-8	0.9999992	0.9999998
Given time-varying covariates	46084	0.9999997	6.7917506E-8	0.9999997	9.1473164E-8	0.9999992	0.9999998
36 months							
Probability of having observed antidepressant medication history							
Given baseline covariates	45235	0.86	0.09	0.88	0.10	0.24	0.99
Given time-varying covariates	45235	0.86	0.09	0.88	0.10	0.20	0.99
Probability of being uncensored							
Given baseline covariates	45235	0.999971	6.6963806E-6	0.999971	9.041897E-6	0.99992	0.99998
Given time-varying covariates	45235	0.999971	6.7459345E-6	0.999971	9.0819261E-6	0.99992	0.99998

## Abbreviations

WHI-CT: Women’s health initiative clinical trials cohort
WHI-OS: Women’s health initiative observational cohort
WHI: Women’s Health Initiative
BMI: Body mass index
IPTW: Inverse probability of treatment weights
CES-D: Center for Epidemiological Studies Depression Scale
PH: Cox Proportional Hazards
CI: Confidence interval
HR: Hazard ratio
MSMs: Marginal structural models
